# A Review of Methods for Sensing the Nitrogen Status in Plants: Advantages, Disadvantages and Recent Advances

**DOI:** 10.3390/s130810823

**Published:** 2013-08-16

**Authors:** Rafael F. Muñoz-Huerta, Ramon G. Guevara-Gonzalez, Luis M. Contreras-Medina, Irineo Torres-Pacheco, Juan Prado-Olivarez, Rosalia V. Ocampo-Velazquez

**Affiliations:** 1 Ingeniería de Biosistemas CA, División de Estudios de Posgrado, Facultad de Ingeniería, Universidad Autónoma de Querétaro, Cerro de las Campanas S/N, 76010 Querétaro, Qro., Mexico; E-Mails: ramon.guevara@uaq.mx (R.G.G.-G.); mcontreras@hspdigital.org (L.M.C.-M.); irineo.torres@uaq.mx (I.T.-P.); 2 Departamento de Ingeniería Electrónica, Instituto Tecnológico de Celaya, Av. Tecnológico y García Cubas S/N, 38010 Celaya, Gto., Mexico; E-Mail: juan.prado@itcelaya.edu.mx

**Keywords:** crop nitrogen, instrumentation, chlorophyll, remote sensing, electrical impedance

## Abstract

Nitrogen (N) plays a key role in the plant life cycle. It is the main plant mineral nutrient needed for chlorophyll production and other plant cell components (proteins, nucleic acids, amino acids). Crop yield is affected by plant N status. Thus, the optimization of nitrogen fertilization has become the object of intense research due to its environmental and economic impact. This article focuses on reviewing current methods and techniques used to determine plant N status. Kjeldahl digestion and Dumas combustion have been used as reference methods for N determination in plants, but they are destructive and time consuming. By using spectroradiometers, reflectometers, imagery from satellite sensors and digital cameras, optical properties have been measured to estimate N in plants, such as crop canopy reflectance, leaf transmittance, chlorophyll and polyphenol fluorescence. High correlation has been found between optical parameters and plant N status, and those techniques are not destructive. However, some drawbacks include chlorophyll saturation, atmospheric and soil interference, and the high cost of instruments. Electrical properties of plant tissue have been used to estimate quality in fruits, and water content in plants, as well as nutrient deficiency, which suggests that they have potential for use in plant N determination.

## 1. Introduction

After carbon, hydrogen and oxygen, nitrogen (N) is one of the essential elements in plants due to its key role in chlorophyll production, which is fundamental for the photosynthesis process. In addition, nitrogen is part of various enzymatic proteins that catalyze and regulate plant-growth processes [1]. Furthermore, nitrogen contributes to the production of chemical components that protect against parasites and plant diseases [2]. Finally, crop yield and biomass are highly affected by N fertilization [3].

Plants absorb nitrogen as a mineral nutrient mainly from soil, and it can be may come in the form of ammonium (NH_4_^+^) and nitrate (NO_3_^−^) [4]. However, soil N supply is often limited [5], which forces farmers to increase the amount of N fertilizers in order to achieve better crop yield. However, farmers may provoke nitrogen overfertilization, which thwarts optimum plant productivity [6], as plants are not able to absorb the excess of N-fertilizer. This entails unnecessary expenditure on the part of farmers. Nitrate leaching, soil denitrification, and volatization are the main processes for N-fertilizer excess loss, contributing to environmental pollution [7]. Nitrate leaching contaminates groundwater and other bodies of water, which may contribute to eutrophization. In addition, volatized N contributes to global warming by releasing nitrous oxides (*i.e.*, NO, N_2_O), which are considered greenhouse gases. Furthermore, a reaction between ammonium ions near the soil's surface and alkaline rainwater generates gaseous ammonia, which disperses into the atmosphere [8]. When there is a high N supply in leafy vegetable crops, N mobile form concentrations (*i.e.*, nitrate, ammonium) increase in leaves, thus becoming hazardous to human health. A high-nitrate diet is an important factor in the development of several human diseases such as methahemoglobinaemia, and gastric and bladder cancer [9].

**Figure 1.**
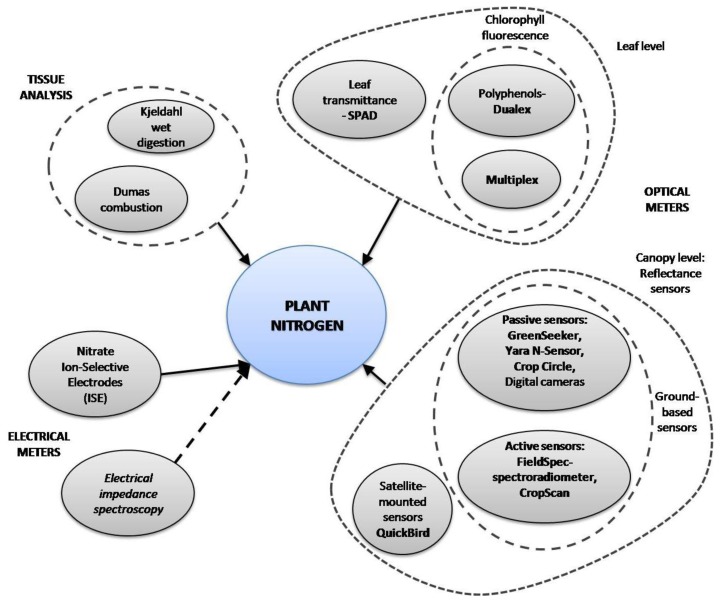
Methods for plant nitrogen sensing.

The main way to optimize farming practices for environmental-friendly yields and to reduce costs is by applying precision agriculture [10], which requires the efficient supply of water and nutrients. Various methods proposed in several studies to optimize fertilization strategies in crops are based on commercial and custom-developed tools. A summary of methods for N determination is shown in Figure 1. Methods based on tissue analysis, such as Kjeldahl-digestion and Dumas-combustion, have been widely applied to plants due to their reliability in organic nitrogen determination, but they are time-consuming and destructive. Several studies have reported on faster and non-destructive new tools designed for plant N status estimation. Optical properties of some leaf pigments, such as chlorophyll and polyphenols, have been used as plant N status indicators [11]. Some of these tools measure leaf chlorophyll content, which is highly correlated to plant N status (*i.e.*, SPAD-502, Dualex, Chlorophyll fluorescence). Remote sensing methods have been developed and implemented to estimate crop N status in a specific area or in the entire field. There are some commercial ground-based active-mounted (*i.e.*, Yara N-Sensor, GreenSeeker, CropScan) and satellite-mounted sensors (*i.e.*, QuickBird), all of which measure crop canopy reflectance in the visible and/or IR wavebands. Some researchers have recently developed new systems based on digital image processing using digital cameras [5,12].

Most of those techniques have some drawbacks, for example: satellite imagery turnaround is time-consuming and weather conditions may interfere with the imagery; ground-based sensors may be affected by soil and light conditions; chlorophyll sensors fail to detect overfertilized plants due to chlorophyll saturation. Apart from the optical properties of leaves, electrical properties of plant tissue can be affected by the plant's nutrition status. The electrical properties of plant sap can be modified by variations in nitrate ion content, which can be measured by using an Ion-Selective Electrode (ISE). Resistance and capacitance are also electrical properties of plant tissue, and their combined effects constitute its electrical impedance. Electrical impedance spectroscopy has been used by a few authors to detect water stress [13], fruit quality [14], and nutrition status [15,16]. The objective of this article is to give an overview on the most used and most recent crop nitrogen sensing methods, techniques and instruments.

## 2. Tissue Analysis: Kjeldahl Digestion and Dumas Combustion

Plant tissue analysis has been used as a reference technique to estimate plant N status. Numerous studies reported the use of plant tissue analysis to compare actual plant N content with plant N status predicted by recent developed systems. Tissue analysis methods are applied to Nitrogen Nutrition Index determination, which is related to crop biomass. Because of their importance in plant N status determination, this section is focused on describing and on the current state of the art of tissue analyses methods.

**Figure 2.**
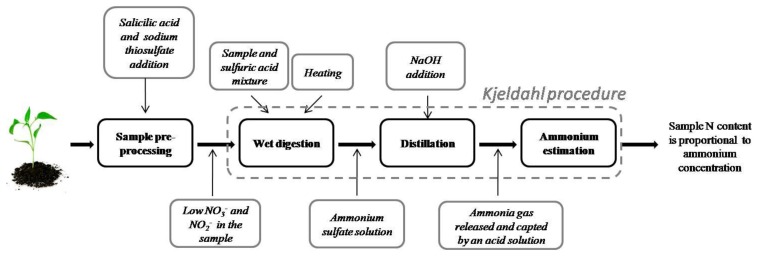
Kjeldahl wet digestion procedure.

One of the most used methods for determining nitrogen in organic compounds was proposed by Johan Kjeldahl in 1883 [17]. This method is known as Kjeldahl digestion, and it has been widely used for nitrogen determination on food, beverages, meat, feed, grain, manure, waste water, soils, and plant tissue. It is considered a method of reference for biological sample nitrogen estimation [18–21]. A Kjeldahl digestion procedure diagram is shown in Figure 2. This method can be roughly broken down into three steps:

1 Wet digestion—A sample is mixed with a proportional amount of a concentrated acid (usually sulfuric acid) in a Kjeldahl flask. The resulting mixture is heated until it clarifies as CO_2_ evolves. Heat input should be as high as necessary to bring 250 mL of water at 25 °C to a rolling boil in 5 min. The end result is an ammonium sulfate solution.
2 Distillation—The Kjeldahl flask is attached to a water condenser. An amount of NaOH is added to the resultant digestion solution to turn ammonium ions (in ammonium sulfate) into ammonia. The solution is heated to release ammonia gas, which passes through the condenser, and then it is trapped in a receiving solution (boric acid, standard acid (HCl) or sulfuric acid) contained in a flask.
3 Ammonium estimation—Due to the fact that ammonia concentration is proportional to nitrogen content in the sample, good ammonia determination is important. The most popular method is titration. Michelowsky and collaborators [22] analyzed the regents used in titration.

In order to accelerate digestion reactions, some other reagents are used. Potassium sulfate is added to the digestion mixture to increase the boiling point, which reduces time digestion. Catalysts are added to accelerate organic compound decomposition such as mercuric oxide, a copper sulfate-titanium oxide combination, or selenium. Kjel-tabs may contain one of these catalysts together with potassium sulfate.

The Kjeldahl digestion method has some disadvantages. This method only measures nitrogen bound to organic components (proteins, amino acids, nucleic acids) and ammonium in the sample. Other nitrogen forms, such as nitrate and nitrite, cannot be measured through this procedure. Biological samples can be processed before digestion in order to reduce nitrate and nitrite to ammonium. Lee and collaborators [23] compared the standard Kjeldahl method with three modified Kjeldahl procedures: the addition of salicylic acid prior to digestion, the pre-reduction of nitrate to ammonium using CrK(SO_4_)_2_, and the addition of phenyl-acetate to the standard digestion mixture. This last procedure yielded the best nitrogen measuring results in plant tissue, but the salicylic acid method performed better in the presence of water. Amin and Flowers [24] reported the use of salicylic acid dissolved in concentrated sulfuric acid to recover other nitro compounds. A Kjeldahl method guide [18] suggests the use of salicylic acid followed by sodium thiosulfate for nitrate reduction.

Some researchers have proposed different techniques for ammonia determination in addition to titration, which is time-consuming [25]. Clifton and Clifton used the indophenol colorimetric method for ammonia determination to apply Kjeldahl digestion to East African savannah grass analysis [26]. Colorimetry was one of the most used methods for ammonia detection as reported by Handson and Shelley [27], and it is also used in the Technicon Auto-Analyzer method. The latter uses continuous flow injection for the extraction of ammonia and indophenol colorimetry for its determination [24]. However, Pontes and collaborators [28] preferred ion chromatography to avoid the carcinogenic reagents used in the indophenol colorimetry method, and to achieve better sensitivity. Although ion chromatography performs better than indophenol colorimetry, it can be considerably expensive, due to the high cost of ion exchange columns and their rapid deterioration [27]. Diffusion conductimetry is the ammonia determination method with the best performance compared to colorimetry and titration [23,25].

Further modifications made by researchers have improved the digestion process. Studies have reported the use of ultrasound and microwave energy in the Kjeldahl procedure. As concluded by Domini and collaborators [20], a combination of ultrasound and microwave energy in the digestion process improves its performance by reducing digestion time to 7 min (compared to 30 min for classical Kjeldahl). Ultrasound energy has been used to substitute the distillation system in classical Kjeldahl for a purge-and-trap system in order to stimulate a chemical reaction between the alkaline reagent and the digestion mixture, as proposed by Pontes *et al.*[28].

**Figure 3.**
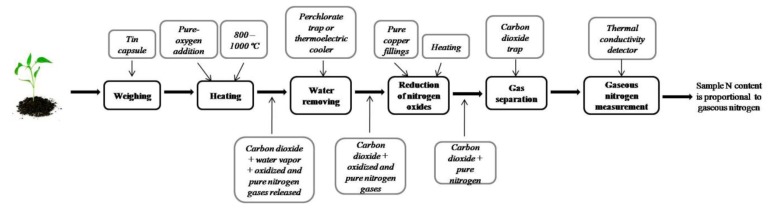
Dumas combustion procedure.

Although researchers have made improvements to the classical Kjeldahl method, one of the most important disadvantages is that Kjeldahl only determines organic nitrogen bound in the trinegative state [28], and cannot measure other nitrogen forms such as nitrate and nitrite. The combustion method for total nitrogen determination proposed by Jean-Baptiste Dumas in 1831 [29] overcomes some Kjeldahl method deficiencies, and it is one of the reference methods for nitrogen determination in organic and inorganic samples. Shown in Figure 3, the Dumas combustion procedure is described as follows:

1 Weighing. Sample is weighed into a tin capsule.
2 Heating. Sample contained in the tin capsule is heated at temperatures ranging between 800–1,000 °C. Pure oxygen is added to accelerate combustion.
3 Water removal. Some gases, such as carbon dioxide, oxygen O_2_, nitrogen oxides, gaseous nitrogen, and water vapor, are generated as combustion by-products. Water vapor is removed either via a perchlorate trap [30] or by passing gas products through a thermoelectric cooler [23] or some other water removal method.
4 Oxide reduction. Nitrogen oxides are reduced to turn them into gaseous nitrogen N_2_. This can be done by passing gasses through pure copper fillings in a reduction heater.
5 Gas separation. Remnant gases, such as carbon dioxide and gaseous nitrogen, are separated and CO_2_ is trapped out to measure only the gas nitrogen concentration.
6 N_2_ measurement. The last step is to measure gaseous nitrogen concentration. A thermal conductivity detector is used in which differences in thermal conductivity of the gases are detected.

As the final product obtained after combustion procedure is N_2_ instead of ammonia, and combustion does not require toxic reagents, the Dumas method is less polluting than Kjeldahl. Additionally, the Dumas technique can determine total nitrogen in the sample better than the Kjeldahl digestion when there is a substantial amount of nitrate-N [31]. Despite advantages regarding the Kjeldahl method, Dumas has some drawbacks. Incomplete combustion causes loss of nitrogen in the sample [30], and the requirement of a small sample weight (200–300 mg) [31] are some of those disadvantages.

## 3. In-Field Systems

In spite of the effectiveness of plant tissue methods, they require invasive sampling of the entire plant or plant parts [32,33]. Moreover, Kjeldahl and Dumas methods require sample preprocessing and analysis in specialized laboratories, which is time-consuming and costly. On the other hand, the toxic reagents Kjeldahl digestion requires and the residual ammonia are harmful to users, as seen. In recent years, several researchers have focused on the design and application of in-field non-invasive methods for use in crop N status determination. Most of these methods work depending on optical plant properties, which are affected by several factors: water content, leaf senescence, diseases, plant nutrients and plant N status [7]. Systems have been developed based on transmittance properties of leaves (SPAD), leaf chlorophyll fluorescence (Dualex), as well as canopy reflectance measurement systems (GreenSeeker, Yara N-Sensor, CropScan), satellite imagery data (QuickBird), and recently, digital image processing. Just as plant optical properties change with nutritional variation, their electrical properties are affected by physiological and nutritional status.

### 3.1. Leaf Chlorophyll Meters

Chlorophyll is the most important pigment in leaves, and it is responsible of their greenness. Leaf chlorophyll content can be used as a nitrogen status indicator because this is an essential element in photosynthetic protein synthesis [4,11]. The SPAD-502-Soil-Plant Analyses Development chlorophyll meter is one of the most used instruments in plant N determination studies [11,34–36]. A leaf section is enclosed in a small chamber and it is exposed to two light sources: (1) a red- (640 nm) and (2) an infrared light (940 nm) positioned just above the leaf. Light filtered through the leaf is captured sequentially by sensors below the leaf. The difference in transmission of the filtered wavelengths is the chlorophyll content indicator per unit leaf area [11]. The Dualex is another device which focuses on measuring polyphenolic compound content in leaves by means of chlorophyll fluorescence. Essentially water-soluble glycosylated flavonoids, polyphenols are carbon-based compounds and secondary metabolism products in leaves, generated when plants are under N deficiency stress and stored in cell vacuoles [11]. They play an important role in plants as chemical defense against herbivores, as well as protection against UV radiation, free radicals, stress, and pathogens [3]. The Dualex measures polyphenols by means of chlorophyll fluorescence [32]. It has two excitation wavelengths: (1) a chlorophyll fluorescence excitation light source at 375 nm (UV), and (2) a reference light source at 650 nm (red). These beams are activated sequentially. UV light is absorbed by polyphenols according to their concentration, and the red light passes through the epidermis without being absorbed before reaching the chlorophyll in the mesophyll. Fluorescent light emitted at 695 nm by chlorophyll excited with UV and red light sources is measured by the Dualex, which calculates a leaf-area dependent ratio between both fluorescence responses.

Because of their portability, quick response, and affordable cost [36], numerous recent studies have focused on utilizing chlorophyll readings to estimate N status in a variety of crops. However, plant growth stage, cultivars, soil water and deficiency of nutrients other than N can affect chlorophyll measurements. Several methodologies have been proposed in those studies to obtain reliable measurements to estimate crops N status. SPAD chlorophyll readings are better when they are taken around the midpoint of a leaf, avoiding the midrib [6,36]. Lin and collaborators [36] reported better results obtained in rice when SPAD readings were taken at 30 mm to either side of midrib, and approximately one-third of the way down from the leaf tip. In order to standardize the N diagnosis procedure using SPAD measurements of rice leaves, they calculated four indices from averaged SPAD chlorophyll readings obtained from the first and the third fully expanded leaves. These indices include: the relative positional difference index between the first- and the third- fully-expanded leaves (RDSI), the normalized difference SPAD index between the first and the third fully-expanded leaves (NDSI), the SPAD ratio of the first and the third fully-expanded leaves (RSI), and the SPAD difference between the first and the third fully-expanded leaves (DSI). Those indices were significantly and exponentially related to leaf N content, and they minimized chlorophyll sensitivity to growth stages and genotypes. In a study focused on leaf N content estimated in ornamental plants, SPAD reading variations due to leaf age can be reduced by choosing the youngest leaves that have already reached 75% of their final growth size [11]. Chlorophyll measurements can be applied in rice under alternate wetting-drying irrigation as well as in continuously flooded fields. Cabangon and collaborators [37] were able to determine crop N status in rice under alternate wetting-drying irrigation without affecting the correlation between leaf chlorophyll and leaf N content. They obtained this result by averaging SPAD readings obtained at 6 randomly selected topmost fully expanded leaves, and irrigation applied when the soil water potential reached—10 kPa at 15-cm depth. They determined a SPAD value of 38 as a critical value to apply N fertilization. In addition to considering where in a leaf SPAD readings should be taken, sunlight exposure should be taken into consideration due to its impact on leaf mass per area, which directly affects leaf N content readings. This was taken into consideration by [6], when they estimated leaf chlorophyll content in nectarine trees. They estimated leaf N content by taking SPAD chlorophyll readings from six leaves located in the mid-section of annual shoots located on the periphery of the tree canopy. This light exposure factor was taken into consideration by Demotes-Mainard and collaborators [11], when they estimated leaf N content in ornamental plants. However, Goffart and collaborators [32] reported that the influence of the time of day in chlorophyll readings taken from potato plants was low, particularly between 9 a.m. and 3 p.m. SPAD chlorophyll meters can only measure leaf N content in one leaf at a time. However, a study developed by Perry and Davenport [34] on apple trees under four different N treatments demonstrated that it is possible to predict N status in surrounding trees under the same N treatment in order to predict applied N. By taking SPAD readings from 24 leaves collected from a plot of 20 trees, averaging and using linear models, an estimated difference of 25 kg·ha^−1^ was obtained between applied N and estimated N.

The N sufficiency index (NSI) has been applied to improve chlorophyll meter ability to detect N deficiencies in several crops, such as cabbage (*Brassica oleracea* spp. capitata), onion (*Allium cepa*), carrot (*Daucus carota*) [3], and mainly in corn (*Zea mays* L.) [38–40]. The NSI is a ratio between chlorophyll readings obtained from plants to be evaluated and well-N fertilized plants. NSI value detected below a threshold of 95% in corn indicates crop N stress and additional N is required [38], whereas a 97% NSI value was established as the threshold by Yu and collaborators [40]. A range between 80% and 100% has been used to make nitrogen recommendations, but a better understanding of the N reference plot, such as applied N source, soil characteristics and application layout is needed [3]. Unlike NSI, a decision rule was applied on potato crops [41] fertilized with 70% of recommended N in order to determine if it is necessary to apply the remaining 30%, based on readings taken from a zero-N reference plot. Another index reported in recent studies is based on a ratio between SPAD chlorophyll and Dualex polyphenolics measurements. The SPAD/Dualex ratio combines the effect of N fertilization on photosynthetic proteins (N-based leaf components) and polyphenols (carbon-based components). Whereas chlorophyll content increases with N fertilization, polyphenolic content is reduced. The SPAD/Dualex ratio showed better response to N fertilization in corn fields, as well as low sensitivity to soil-water content variations [42]. As reported by Demotes-Mainard [11], SPAD readings were less reliable at predicting leaf N content in woody plants, whereas Dualex was able to measure it more precisely. However, the SPAD/Dualex ratio was more sensitive to leaf N content in woody and ornamental plants because it can detect leaf chemical composition changes rather than the effect of dry mass dilution caused by treatments or plant aging [11].

Researchers have developed methods and sensors capable of measuring leaf chlorophyll fluorescence in order to determine plant stress [43]. Indeed, this principle has been used to estimate plant nitrogen status, as reported by Thoren and Schidhalter [44]. Unlike the Dualex, the system used by these researchers was able to take measurement at 3–4 m distance between the sensor and plants. A 630 nm laser was used whose beam was pulsed in order to separate sunlight fluorescence from laser fluorescence. The sensor detected radiation emitted by chlorophyll by means of a specialized optic telescope with beam splitters and filters at wavelengths of 690 and 730 nm. The N content of the plants was determined by calculating the fluorescence ratio of the abovementioned wavelengths. As reviewed by Tremblay and collaborators [45], determining N status in plants by means of chlorophyll fluorescence can overcome some of the limitations of reflectance-based chlorophyll methods. Fluorescence sensors can eliminate erroneous signals from bare soil, but they cannot be used in the same way as other remote sensors, such as Yara N-Sensor, GreenSeeker™ RT 200, or the Crop Circle ACS-430, all based on reflectance measurements. Multiplex^®^ is a more recently developed hand-held optical fluorescence sensor. It excites chlorophyll by generating four wavelenghts (UV-A (375 nm), Blue (450 nm), Green (530 nm), and Red (630 nm)), and detecting chlorophyll fluorescence at different bands, such as: (a) at blue-green (447 nm) when blue excitation is not used, or at yellow (590 nm) when it is, (b) at red (665 nm), and (c) far-red (735 nm). This sensor can overcome a chlorophyll meter deficiency because it is able to distinguish N treatments equally well in shadow or full sunlight, and at any time during the day [45]. However, because the Multiplex sensor requires measurements made approximately 10 cm apart from the plant, covering a 50-cm^2^ area, it is difficult to apply this sensor in monitoring N status in larger areas. Despite this, in recent studies there has been reported Multiplex sensor applied to determine crop N status as a ground-based sensor [45,46].

### 3.2. Canopy Data Processing Systems

Plant N status has been assessed by using non-invasive remote sensing methods which measure the canopy reflectance of a specific area or the entire crop field. Furthermore, leaf chlorophyll readings lack the sensitivity to distinguish between different N treatments [34]. Several studies have focused on crop N assessing by processing canopy reflectance images. It is possible to process reflected electromagnetic energy, and several plant biochemical and biophysical properties can be estimated, such as leaf area index (LAI), aboveground biomass (AGB), and N concentration [47]. In addition, other indices have been calculated based on certain waveband combinations of canopy reflectance data, such as normalized difference vegetation index (NDVI), ratio vegetation index (RVI), and other indices, calculated predominantly with red, green and near-infrarred reflectance data of a crop canopy.

Commercial reflectance sensors applied to estimate crop N status can be classified as passive and active, depending on their light source. Passive crop canopy reflectance sensors measure crop canopy reflectance provided by sunlight. As described by Li and collaborators [48], the hand-held FieldSpec spectro-radiometer sensor (Analytical Spectral Devices, Inc., Boulder, CO, USA) is a hyperspectral device that has 512 channels, capable of measuring in the 325–1,075 nm range (visible and NIR wavelength ranges) of solar light reflected by the assessed canopy. In their study, spectral indices based on differences (NDVI, green NDVI, red NDVI, red and green vegetation index [RGVI]), and others based on ratios (red vegetation index [RVI], green vegetation index [GVI], red and green vegetation index) were calculated from averaged crop canopy reflectance readings at green (520–600 nm), red (630–690 nm), and NIR (760–900 nm) bandwidths. A positive linear relationship between RVI and N uptake in winter wheat was demonstrated, as well as insensitivity to growth stages and crop varieties. In addition, RVI could estimate N status in overfertilized fields. A spectral range from 350 to 2,500 nm with 4 nm of spectral resolution and a 1nm sampling step was applied to estimate plant nitrogen concentration in paddy rice [47] by means of normalized difference indices from the combination of all possible reflectance measurements within that range. The best correlation between plant nitrogen concentration and a normalized difference index was obtained by using reflectance data in the visible part of the spectrum (503 and 480 nm). The FieldSpec spectro-radiometer required a calibration procedure carried out by measuring reflectance of a dark and a white reference panel with known reflectance properties [48]. A recent study [49] reports a comparison between leaf pigment analysis and chlorophyll-based indices calculated from leaf reflectance data measured with the abovementioned spectroradiometer in switchgrass fields under different N treatments. Reflectance data measured at three different wavelengths (500, 670, 700 nm) was used to calculate the Transform Chlorophyll Absortion Ratio Index (TCARI), which was the best correlated index to chlorophyll content (negative correlation in the 10–70 μg/cm^2^ range; positive below 10 μg/cm^2^). CropScan is another passive reflectance sensor that measures incident and reflected light canopy radiation at several wavelength bands from IR to UV [32]. Despite CropScan data being sunlight dependent, this instrument is able to take readings with a larger number of wavelengths compared to an active sensor such as the GreenSeeker, which is less sensitive to weather conditions [50]. This CropScan property allows choosing reflectance wavelengths to calculate vegetation indices that are better suited to crop N level. In the study developed by Zhu and collaborators [51], sixteen wavebands in the 447–1,752 nm range were measured with the CropScan reflectance sensor from rice and wheat fields to correlate their canopy reflectance with leaf N accumulation. They concluded that good correlation between reflectance and leaf N accumulation was obtained in at single wavebands centered at 810 nm and 870 nm, but a ratio of reflectance at 810 nm to reflectance at 660 nm and a ratio of reflectance at 870 nm to at 660 nm had a better response. Studies have also reported reflectance measurements obtained by using a LI-COR spectroradiometer. A simple index based on the first derivative of canopy reflectance spectrum at 735 nm was developed by Lee and collaborators [52]. A LI-1800 (LI-COR Inc., Lincoln, NE, USA) was used to obtain canopy reflectance spectra within the 400–1,100 nm wavelength range with a 10 nm sampling step, in fields with rice in the panicle formation stage. The first derivative was calculated by subtracting reflectance at 730 nm from reflectance at 740 nm and dividing by 10 nm. A combined use of the Field Spec and a LI-1800 spectroradiometers was applied to detect over-fertilized rice plants [53]. A modified simple ratio index was proposed and calculated by dividing the subtraction between reflectance readings at 750 nm and at 445 nm by the subtraction between reflectance readings at 705 nm and at 445 nm. This index was calculated using reflectance readings taken on the first and the third uppermost leaves in rice at panicle initiation. The use of the subtraction result of the index values obtained from the first and the third leaf was particularly effective in the detection of overfertilized N rice plants, in addition to being characterized by low sensitivity to growth stages and genotypes.

Unlike passive reflectance sensors, active sensors have their own light source, thus sunlight dependence is avoided. A list of commercial systems based on active sensors, whose use at field scale is reported in recent studies, is presented as follows: (a) the tractor-mounted Yara N-Sensor (Yara International ASA, Oslo, Norway), (b) the hand-held GreenSeeker (NTech Industries Inc., Ukiah, CA, USA), and (c) the hand-held Crop Circle (Holland Scientific, Lincoln, NE, USA). The Yara N-Sensor has a Xenon-flashlamp, which provides high intensity multispectral light, can measure and record the crop light reflectance in a waveband between 450 and 900 nm [32]. There is another spectrometer inside this instrument that captures ambient light, which is used for permanent correction of the crop reflectance signal [54]. The other active reflectance sensors only generate two wavelengths: a red (660 nm) and a NIR (770 nm) used by the GreenSeeker, whereas the CropCircle has an amber (590 nm) and a NIR (880 nm) light generator [55]. Tremblay and collaborators [54] reported a comparison between the performance of Yara and GreenSeeker sensors was done by analyzing NDVI values determined by these instruments at specific growth stages in corn and spring wheat fields. The Yara sensor is capable of measuring and recording spectral information in a wider range than GreenSeeker. The Yara N-Sensor recorded spectral information at five single wavebands, from which data collected at 660 and 740 nm was used for calculating NDVI. For wheat, NDVI values calculated by GreenSeeker and Yara were able to describe the variation in the crop canopy according to the crop's N status, but Yara was less sensitive than GreenSeeker when NDVI values were close to saturation levels. For corn, the study concluded that both sensors may be used to derive crop N requirements before the V5 growth stage. The Yara N-sensor captures more biomass per unit of soil surface than GreenSeeker, and it is able to be configured to calculate indices such as NDVI and other ratio-based indices, but its performance relies on sunlight conditions, so its use was limited to daytime hours. Although only NDVI was calculated in this study, GreenSeeker is able to compute visible and NIR reflectance data, such as simple ratio (NIR/VIS), inverse ratio (VIS/NIR), and soil adjusted vegetation index, which also requires a soil brightness factor [55]. Similarly to the abovementioned study, an effectiveness comparison between CropCircle and GreenSeeker was reported by Shaver and collaborators [55] based on NDVI calculated by each sensor using reflectance data taken from maize fields. Both sensors performed well in the determination of N variability in irrigated maize at the V12 and V14 growth stages. However, they suggest that the GreenSeeker could reach saturation earlier in the growing season than the CropCircle, so GreenSeeker could not be used when plant biomass was larger at later growth stages.

Another method used to assess N status in a crop field is by taking satellite images from space-mounted sensors, which can deliver information for N management over a larger region [12]. In fact, those satellite-mounted sensors are able to provide detailed information with-in field variability [32]. QuickBird is one of the most used satellite sensors in crop N assessing because the green (520–600 nm), red (630 nm), and NIR bands (760–900 nm) are important for discriminating water and N deficiency [56]. Image data from QuickBird has high spatial resolution (0.61 for panchromatic and 2.44 m for multispectral images at nadir). Despite their advantages, there are some drawbacks, such as: (a) high cost; (b) interference owing to weather conditions (*i.e.*, cloud interference); (c) a slow turnaround in image pre-processing, which may require considerable processing time; and (d) delays between image capture and the availability of usable data [12,32,56]. Although, other satellite sensors such as Ikonos, Hyperion, and CHRIS/Proba, have been recently used for chlorophyll content estimation [57]. More accurate N deficiency detection could be reached in near future because of the current development of hyperspectral sensors [32]. As an example, several new hyperspectral missions including Hyper-X, EnMap, HERO, and HyspIRI, are emerging [58]. In addition, new satellite sensors, such as RapidEye, WorldView-2, and SumbandilaSAT include off-chlorophyll absorption wavebands [57].

Recent research has focused on the use of digital imaging methods for crop N estimation. Several stress and biophysical plant parameters can be detected with digital imaging, for example: tiller densities across a field, insect damage, and nutrient and water deficiencies [59]. Unlike most remote sensing methods, digital imaging does not require sophisticated instruments (*i.e.*, spectroradiometers, Crop Canopy reflectance meters), but only a commercial digital camera and an image processing system. The system proposed by Kim and collaborators [60] used a custom-developed 3-CCD camera as a multi-spectral imaging sensor, while a high-resolution Leica S1 PRO digital imager was employed by [59]. A custom system based on a pushbroom CCD camera was designed in order to acquire hyperspectral images of wheat canopy [5]. Li and collaborators [12] designed their own digital imager by using a CMOS camera built into a hand-held computer combined with a GSM mobile telephone. Different techniques have been applied in these digital image processing methods. Images of broccoli were taken with a digital camera in conjunction with a daylight long-pass filter, which cut reflectance spectra detected at 780 nm, and processed using CIELab color space in Adobe Photoshop 5.0 software. As a result, the CIELab “b” parameter in the wavelength ranges of 510–780 nm and 516–780 nm were highly correlated to N status [59]. A criterion of greenness, derived from green and red reflectance, as well as a soil adjustment, was determined in order to estimate the proportion of pixels that met with it. A Canopy Cover index was calculated based on that proportion, and a close correlation was found with Canopy Cover values and NDVI from GreenSeeker and the Yara N-Sensor [12]. Vigneau and collaborators [5] proposed a new method to estimate the NNI through LAI and chlorophyll content in wheat by processing digital images and obtaining reflectance spectra (400–1,000 nm) of whole plants. Digital image processing was applied to tomato seedlings in order to estimate their N status, which is difficult to measure by means of SPAD [61]. A Sony cyber-shot commercial digital camera was used to take images of tomato plants, which were under a light-controlled environment, and an analysis using RGB color space was carried out. It was demonstrated that this method could overcome variability due to growth conditions, compared to the SPAD chlorophyll meter. By combining a reflectance correction and pre-processing (data normalization and centering), SPAD readings had low sensitivity to leaf inclination and specular reflection. Image quality must be maintained for proper spectral image analysis, but it can be affected by changes in ambient light, so Kim and collaborators [60] developed a fuzzy logic control algorithm to automatically adjust the camera exposure and gain in order to control image brightness within a target gray level.

### 3.3. Nitrate Sap Content and Electrical Variables

Plant nutritional and physiological status affects plant reflectance, absorbance and transmittance measurements. However, plant N optical sensing methods have some deficiencies as they are susceptible to sunlight variation, soil conditions, and chlorophyll saturation, among other types of interference, which does not allow the detection of overfertilized plants. Furthermore, SPAD chlorophyll readings do not respond as rapid as petiole nitrate sap concentration when detecting plant N deficiency. In potato crops, chlorophyll readings were able to detect severe N deficiency about one month after emergence, whereas petiole nitrate sap concentration measurements responded within two weeks [56]. According to Parks and collaborators [9], chlorophyll readings were not able to distinguish between N supply treatments in sugar beet until 61 days after planting, and 108 days in broccoli, whereas petiole sap nitrate concentration could distinguish N treatments after 47 and 87 days respectively. Plant nitrate sap concentration is commonly used in crop N status determination because it is closely correlated to plant N status. Two types of instruments are reported in several studies: the nitrate Ion Selective Electrode (ISE), and the combination of nitrate test strips and a hand-held reflectometer [32]. Merkoquant test strips can measure up to 500 mg·L^−1^, whereas Reflectoquant strips are capable of measuring up to 225 mg·L^−1^. Samples of plant sap can be obtained generally from any fleshy petiole [41], but a petiole could be chosen depending on the crop: from artichoke leaves that have recently matured [62], from stem bases of winter wheat or maize [63], or from the leafstalks of the fifth or the sixth external completely developed leaf in lettuce [64]. Nitrate test strips turn their two-reactive zones into a red-violet combination when exposed to nitrate contained in the sample, and that color change can be measured by a hand-held reflectometer. Two commercial hand-held reflectometers are reported in studies: the Nitrachek [32,64] and the RQflex [9,62]. Also, plant sap electrical conductivity can vary according to its Nitrate-N content, among other ions, and an Ion Selective Electrode (ISE) can determine plant sap nitrate concentration. An example of nitrate ISE is the ‘TwinNO_3_^−^ B-341’ (Horiba, Kyoto, Japan), which is portable for use in-field [9]. This type of electrode has a sensor pad that contains two electrodes, and the sap sample is dropped in it. Voltage is generated between electrodes according to the Nernst equation, and it depends on the nitrate level in solutions. This nitrate ISE has an operational range of 23–2,235 mg·L^−1^.

Test strips are not considered hazardous to humans, and the equipment cost is relatively low [32]. However, the maximum nitrate concentration that test strips can estimate is well below typical concentrations in plant sap, so the latter must be diluted before measuring. Goffart [32] reported that plant sap should be diluted to concentrations lower than 78 mg·L^−1^. Moreover, ISEs tend to be biased, which is attributed to interference from plant organic compounds and other ions such as: chloride, bicarbonate, and nitrite [65]. As suggested by Tremblay and collaborators [3], sap nitrate tests are not widely applied in crop N status determination, due possibly to the sampling, preservation, extraction, dilution, and measurement effort required. Despite these disadvantages, at least 10 lettuce plants [9,64], or 20 petioles from different plants in the entire field, whose measurements take at least 45 min [9,32], are required to estimate an acceptable nitrate concentration. A diagnosis method based on sap nitrate concentration in winter wheat and maize was described by Lemaire and collaborators [63] as follows: (1) the calculation of the overall fertilizer requirements of the crop using the balance-sheet method that gives the total N rates; (2) the application of a reduced amount of fertilizer (X−40 kg·ha^−1^) by splitting application at tillering and at the beginning of stem elongation; (3) the measurement of the nitrate concentration at the base of the main stem to detect N deficiency at three stages during stem elongation (1st node, 2nd node and flag leaf emergence); and (4) the application or omission of the last dressing of 40 kg·ha^−1^ or more depending on the stage at which N deficiency occurs and its intensity. Although this procedure cannot be used to quantitatively estimate crop N status, it could distinguish probable N deficiency situations from situations with N sufficiency.

Just as plant sap has electrical properties due to its ionic content, plant biological tissues can behave as electrical circuits made with passive elements such as resistors and capacitors [66], which together constitute electrical bioimpedance. It is defined as the opposition to the passage of AC signals through an electrical system, which generates amplitude changes and phase shifts between output and input system signals, and it is a frequency-dependent variable. Electrical impedance of biological tissues is a variable that can be affected by various factors, such as water level and diseases. It is used in medicine to estimate total water content in the body [67], body mass index and body fat percentage [68], and cell behavior [69], among others. Likewise, electrical impedance measurements have been applied to plant tissue to monitor the quality of fruit [14] by estimating maturity level, the concentration of soluble solids, the amount of moisture, *etc.* The effects of drying and freezing-thawing processes modify electrical impedance measurements in eggplant pulp [70] as well as water stress in tomato plants [13] have been reported. Other studies also reported correlations between electrical impedance and the concentration of certain nutrients in the plant, such as calcium, phosphorus and potassium [15]. A recent nutrition index was developed and applied to tomato plants stressed by lack of mineral nutrients [16]. Electrical impedance measurements can provide more accurate and timely information about plant physiological and nutritional status because it can respond to changes in cellular structure, membrane potential, and sap ionic concentration more directly than non-contact sensing methods. This suggests that electrical impedance can be used to monitor plant N status as some of those physiological variables are affected by N concentration.

## 4. Summary of Advantages and Disadvantages

After exposing and discussing these techniques for plant N status determination, a summary of them is showed in Table 1. Advantages and disadvantages of these techniques are listed, which are in accordance with studies recently developed and referenced in this review.

## 5. Conclusions

Due to its environmental and economic impact, there is growing concern over N fertilizer management. Kjeldahl digestion and Dumas combustion have been used as reference methods to determine organic and ammonium N contents. However, they are highly destructive and time consuming, require the use of toxic reagents and sample preprocessing.

In addition to being non-invasive, plant optical properties have been applied to measure N status in plants due to the high-correlation between leaf pigments and N content. However, at high N supply, chlorophyll reaches saturation, preventing the detection of excessive N in plants. In addition, optical properties can be affected by other types of stress, such as water deficiency. Despite this, relative values of chlorophyll concentration obtained by using reference N plot within a field (zero N or over fertilized N) aim to alleviate the effect of other factors than N status in crop fields, increasing the sensitivity of the optical measurements. Indices and methodologies have been created in order to achieve better N status measurement. Satellite imagery can be used to estimate crop N status in an entire field, but they are time-consuming because images are not refreshed as quickly as required for N management.

New methodologies involve a combination of techniques, such as measurements of stable (*i.e.*, chlorophyll) and mobile (*i.e.*, ammonium [NH_4_^+^] and nitrate [NO_3_^−^]) N forms. Petiole nitrate concentration has been used in several studies, and it has been demonstrated that plant nitrate concentration measurements are more sensitive to N changes than optical measurements. In addition, sap nitrate concentration can be used for detecting overfertilized plants. However, sap nitrate concentration is not able to measure other N forms. Despite this, nitrate concentration combined to optical measurements can overcome some limitations of both techniques. Plant tissue has electrical properties such as conductivity, resistance, capacitance, among others. It has been demonstrated that electrical impedance applied to biological tissue can be helpful in determining its physiological status. However, electrical impedance has been applied predominately for medical purposes. Few studies have reported the relationship between electrical impedance and plant nutritional status. In spite of this, those few studies have demonstrated electrical impedance can be potentially used to determine plant nutritional status, and more precisely, as a plant N status estimator.

## Figures and Tables

**Table 1.** Advantages and disadvantages of plant N sensing techniques discussed.

**Techniques**				**Advantages**	**Disadvantages**
Tissue analysis			Kjeldahl digestion	Reference method to estimate total N contents (protein, amino acids, nucleic acids, *etc.*).	Invasive and destructive. Time-consuming. Toxic reagents used. Sample preprocessing requirements.
			Dumas combustion	No nitrate and nitrite reduction.	Destructive. Nitrogen loss due to incomplete combustion. Sample preprocessing requirements.
Optical meters	Leaf level	Transmittance	SPAD	Non invasive due to the high correlation between N status and leaf chlorophyll contents. Portability.	Unable to detect overfertilized crops due to the chlorophyll saturation. Low sensitivity for detecting N stress at early stages.
		Fluorescence	Dualex	Can eliminate erroneous signals from bare soil. Distinguish between different N treatments in shadow of full sunlight. Portability. Multiplex is able to detect N deficiency among other stresses (*i.e.*, pathologies, water stress)	They are still not able to be used as ground-based remote sensor. In spite of this, fluorescence sensors could be used for monitoring larger crop areas in the near future.
			Multiplex		
	Canopy level	Ground-based	Passive sensors: FieldSpec CropScan LI 1800	Can detect a greater crop field area than leaf level meters.	Calibration is required. Sunlight dependence.
			Digital cameras	Do not require sophisticated instruments.	Sunlight dependence. Although, recent studies have reported the use of fuzzy logic controllers for reducing sunlight effects, more research focused on crop N status analyses is still required.
			Actrive sensors: GreenSeeker Yara N-Sensor CropCircle	No dependence to sunlight, due to their own light sources. Yara captures more biomass per unit of soil surface, and measures and record a wide waveband. GreenSeeker can describe the variation in the crop canopy according to the crop's N status, even close to N saturation.	Expensive equipment. Saturation due to the biomass increasing. GreenSeeker limitations because it is able to measure only two wavelengths. Yara performance could not be able to detect plant N status when they are close to N saturation. GreenSeeker reaches saturation earlier than CropScan when measuring crop N status during growth stage.
		Satellite-mounted	QuickBird	Allows the entire field analysis.	Expensive imagery. Interferences by atmospheric conditions. Slow turnaround. Despite this, new satellite constallations are coming with higher spectral, spatial and temporal resolutions, improving their ability to determine crop N status.
Sap and electrical meters			Nitrate test strips	High correlation between N status and plant sap nitrate concentration. Cheap and portable. Quick measurements.	Nitrate variations caused by light exposure. Plant sap dilution is required. Destructive. No other N forms are considered, so it is not able to measure total N in plant tissue.
			Nitrate ISE	Due to the high correlation between N status and sap nitrate concentration, N content can be estimated by means of nitrate ions in plant sap. A wider operative range than nitrate strips.	Sensitivity to other ions, such as chloride, bicarbonate and nitrite. Nitrate concentration depends on other variables than N status (*i.e.*, diurnal variation, sampling procedures) Destructive. Calibration is needed. Sap dilutions are required. No other N forms are considered, so it is not able to measure total N in plant tissue.
			Electrical impedance spectroscopy	Direct measurement of plant tissue electrical properties.	Invasive. Electrode polarization effects. There are few studies focused on plant nutrient sensing.
